# Point-prevalence survey of healthcare facility-onset healthcare-associated *Clostridium difficile* infection in Greek hospitals outside the intensive care unit: The C. DEFINE study

**DOI:** 10.1371/journal.pone.0182799

**Published:** 2017-08-16

**Authors:** Athanasios Skoutelis, Angelos Pefanis, Sotirios Tsiodras, Nikolaos V. Sipsas, Moyssis Lelekis, Marios C. Lazanas, Panagiotis Gargalianos, George N. Dalekos, Emmanuel Roilides, George Samonis, Efstratios Maltezos, Dimitrios Hatzigeorgiou, Malvina Lada, Symeon Metallidis, Athena Stoupis, Georgios Chrysos, Lazaros Karnesis, Styliani Symbardi, Chariclia V. Loupa, Helen Giamarellou, Ioannis Kioumis, Helen Sambatakou, Epameinondas Tsianos, Maria Kotsopoulou, Areti Georgopali, Klairi Liakou, Stavroula Perlorentzou, Stamatina Levidiotou, Marina Giotsa-Toutouza, Helen Tsorlini-Christoforidou, Ilias Karaiskos, Georgia Kouppari, Eleftheria Trikka-Graphakos, Maria-Anna Ntrivala, Kate Themeli-Digalaki, Anastasia Pangalis, Melina Kachrimanidou, Maria Martsoukou, Stergios Karapsias, Maria Panopoulou, Sofia Maraki, Anagnostina Orfanou, Efthymia Petinaki, Maria Orfanidou, Vasiliki Baka, Antonios Stylianakis, Iris Spiliopoulou, Stavroula Smilakou, Loukia Zerva, Evangelos Vogiatzakis, Eleni Belesiotou, Charalambos A. Gogos

**Affiliations:** 1 General Hospital of Athens ‘Evangelismos’, Athens, Greece; 2 Sotiria’ General and Chest Diseases Hospital of Athens, Athens, Greece; 3 Medical School, National and Kapodistrian University of Athens, ‘Attikon’ University Hospital, Athens, Greece; 4 Laiko General Hospital and Medical School, National and Kapodistrian University of Athens, Athens, Greece; 5 General Hospital of Attika ‘KAT’, Athens, Greece; 6 General Hospital of Athens ‘Korgialenio-Benakio’ Red Cross, Athens, Greece; 7 General Hospital of Athens ‘G.Gennimatas’, Athens, Greece; 8 University of Thessaly, Medical School, University General Hospital of Larissa, Larissa, Greece; 9 Aristotle University of Thessaloniki, Hippokration General Hospital of Thessaloniki, Thessaloniki, Greece; 10 University of Crete, School of Medicine, University Hospital of Heraklion, Heraklion, Crete, Greece; 11 Democritus University of Thrace, University General Hospital of Evros, Alexandroupoli, Greece; 12 251 Air Force General Hospital, Athens, Greece; 13 Sismanoglion General Hospital of Athens, Athens, Greece; 14 Aristotle University of Thessaloniki, Medical School, AHEPA University Hospital of Thessaloniki, Thessaloniki, Greece; 15 Athens Medical Centre, Maroussi, Athens, Greece; 16 Tzaneio General Hospital, Piraeus, Greece; 17 401 Military Hospital of Athens, Athens, Greece; 18 Thriasio General Hospital of Elefsis, Attica, Greece; 19 A.Fleming General Hospital of Athens, Athens, Greece; 20 HYGEIA Hospital, Athens, Greece; 21 Aristotle University of Thessaloniki, General Hospital ‘G.Papanikolaou’, Thessaloniki, Greece; 22 National and Kapodistrian University of Athens, Hippokration General Hospital, Athens, Greece; 23 University of Ioannina, University General Hospital of Ioannina, Ioannina, Greece; 24 Metaxa Cancer Hospital, Piraeus, Greece; 25 Astellas Pharmaceuticals, Attica, Greece; 26 Hippokration General Hospital of Athens, Athens, Greece; 27 General Hospital ‘G.Papanikolaou’, Thessaloniki, Greece; 28 University Hospital of Heraklion, Heraklion, Crete, Greece; 29 Hippokration General Hospital of Thessaloniki, Thessaloniki, Greece; 30 University of Patras, Medical School, University General Hospital of Patras, Patras, Greece; 31 Laiko General Hospital, Athens, Greece; Cleveland Clinic, UNITED STATES

## Abstract

**Background:**

The correlation of *Clostridium difficile* infection (CDI) with in-hospital morbidity is important in hospital settings where broad-spectrum antimicrobial agents are routinely used, such as in Greece. The C. DEFINE study aimed to assess point-prevalence of CDI in Greece during two study periods in 2013.

**Methods:**

There were two study periods consisting of a single day in March and another in October 2013. Stool samples from all patients hospitalized outside the ICU aged ≥18 years old with diarrhea on each day in 21 and 25 hospitals, respectively, were tested for CDI. Samples were tested for the presence of glutamate dehydrogenase antigen (GDH) and toxins A/B of *C*. *difficile*; samples positive for GDH and negative for toxins were further tested by culture and PCR for the presence of toxin genes. An analysis was performed to identify potential risk factors for CDI among patients with diarrhea.

**Results:**

5,536 and 6,523 patients were screened during the first and second study periods, respectively. The respective point-prevalence of CDI in all patients was 5.6 and 3.9 per 10,000 patient bed-days whereas the proportion of CDI among patients with diarrhea was 17% and 14.3%. Logistic regression analysis revealed that solid tumor malignancy [odds ratio (OR) 2.69, 95% confidence interval (CI): 1.18–6.15, p = 0.019] and antimicrobial administration (OR 3.61, 95% CI: 1.03–12.76, p = 0.045) were independent risk factors for CDI development. Charlson’s Comorbidity Index (CCI) >6 was also found as a risk factor of marginal statistical significance (OR 2.24, 95% CI: 0.98–5.10). Median time to CDI from hospital admission was shorter with the presence of solid tumor malignancy (3 vs 5 days; p = 0.002) and of CCI >6 (4 vs 6 days, p = 0.009).

**Conclusions:**

The point-prevalence of CDI in Greek hospitals was consistent among cases of diarrhea over a 6-month period. Major risk factors were antimicrobial use, solid tumor malignancy and a CCI score >6.

## Introduction

*Clostridium difficile* infections (CDI) have emerged as a major health problem associated with hospitalization [1]. However, in many hospitals, testing frequency remains low due to the absence of clinical suspicion of CDI [2]. Where stool testing is requested, diagnosis often relies on inexpensive, easy–to-perform and rapid enzyme immunoassays with the main disadvantage of low sensitivity [3,4]. The main disadvantage of this assay is the frequency of false negative results and therefore an underestimation of CDI epidemiology. Reliable, rapid detection of CDI is essential for individual patient management, infection control, and to allow a better understanding of CDI epidemiology, which is important for: a) raising awareness of attending physicians to the magnitude of the problem; and b) early detection of patients with probable CDI. This is of great medical importance because CDI can be difficult to treat and the risk for disease recurrence is high even after initial clinical response to treatment [5].

In 2008, a European epidemiological survey reported a weighted mean incidence of healthcare-associated CDI in Europe of 4.1 per 10,000 hospital patient-days [6]. In a more recent European biannual point prevalence study in 2012–2013, the reported mean incidence rate increased by 71%, from 4.1 to 7.0 cases per 10,000 patient bed-days [2]. Specific predisposing risk factors for CDI include administration of broad-spectrum antimicrobials [7–11], increased age [12–14] and underlying immunodeficiency [15]. The risk for CDI is proportionally increased not only with the number of administered antimicrobials, but also with the days of antimicrobial administration [8]. The traditional concept that administration of clindamycin is the most important risk factor for CDI [9–11] has been advanced by more recent data showing that other antimicrobials, such as fluoroquinolones, are also implicated in the development of CDI [8].

The correlation of CDI with in-hospital morbidity [16] is important for hospital settings in which broad-spectrum antimicrobial agents are routinely used. This is a common scenario in hospitals where infections by multidrug-resistant bacteria predominate and the consumption of broad-spectrum antimicrobials is high [17], as in tertiary hospitals in Greece. C. DEFINE is the first national epidemiological study in Greece, and aimed to unravel the prevalence of CDI and explore the predisposing factors linked with the infection. We revealed a considerable point-prevalence of CDI in Greek hospitals in each study period and identified four main risk factors predisposing hospitalized patients in Greece to CDI.

## Patients and methods

### Study design

This was a biannual point-prevalence study conducted in 21 (March 2013) and 25 (October 2013) study sites in Greece; four of the sites participating in October 2013 did not manage to participate in March 2013, due to delay in hospital approvals. The participating hospitals were as follows: University General Hospiital of Athens ATTIKO, Athens, Greece; General Hospital of Athens 'Laiko', Athens, Greece; General Hospital of Athens 'Evangelismos', Athens, Greece; General Hospital of Athens 'G. Gennimatas', Athens, Greece; General Hospital of Athens ‘Korgialenio-Benakio’ Red Cross-Athens, Greece; Tzaneio General Hospital, Piraeus, Greece; Metaxa Cancer Hospital, Piraeus, Greece; General Hospital of Athens 'Ippokration', Athens, Greece; Thriasio General Hospital of Elefsi, Attica, Greece; Sismanoglion General Hospital of Athens, Athens, Greece; University Hospital of Heraklion, Crete, Greece; University General Hospital of Patras, Patras, Greece; Hippokration General Hospital of Thessaloniki, Thessaloniki, Greece; General Hospital ‘G.Papanikolaou’, Thessaloniki, Greece; University General Hospital of Ioannina, Greece; 251 Air Force General Hospital, Athens, Greece; 401 Military Hospital of Athens-Athens, Greece; ‘Sotiria’ General and Chest Diseases Hospital of Athens, Athens, Greece; HYGEIA Hospital, Athens, Greece; Athens Medical Centre, Maroussi, Athens, Greece; AHEPA University Hospital of Thessaloniki, Thessaloniki, Greece; University General Hospital of Larissa, Larissa, Greece; University General Hospital of Evros, Alexandroupoli, Greece; Amalia Fleming General Hospital of Athens, Athens, Greece; and General Hospital of Attika ‘KAT’, Athens, Greece.

The study was conducted after review and approval from the local Ethics Committees of the participating hospitals, and after written informed consent of patients with diarrhea. The study was performed on one day from 8:00 hrs to 18:00 hrs in two different time periods in each hospital. The purpose was to register all cases of diarrhea on the respective study days. The first study period was March 1st to March 31st 2013 and the second study period was October 1st to October 31st 2013. The exact date of participation of each site was selected at the discretion of the attending physicians.

Inclusion criteria for the study among screened patients were: a) written informed consent by the patients, or their legal representatives for patients unable to consent; b) age ≥18 years; c) diarrhea defined as at least 3 episodes of unformed stools in the 24 hours before inclusion (type 5–7 of the Bristol stool chart) [18]. Hospitalized patients with an existing diagnosis of CDI on the study date were also enrolled. CDI was defined as any episode of diarrhea occurring either during hospitalization or within 12 weeks of any hospital discharge with documented presence of toxigenic *C*. *difficile* after investigation by the reference laboratory, as defined below.

Data were collected for patients fulfilling the inclusion criteria from hospitals’ internal medicine, oncology, hematology, gastroenterology, nephrology, pulmonary medicine, radiotherapy, cardiology, surgery, vascular surgery, neurosurgery, orthopedics and urology departments, except for patients hospitalized in intensive care units (ICUs). Patients in the ICU were excluded because ICU patients often present with diarrhea associated with causes other than CDI, and this could introduce confounding factors. The investigators had to report the total number of patients they screened for diarrhea (the total number of hospitalized patients on the study day) in each specific department. The following information was recorded for each patient with diarrhea: a) demographics; b) date of hospital admission; c) starting date of diarrhea; d) vital signs, presence of severe abdominal pain, number of bowel movements 24 hours before study enrolment and white blood cells on the study date; d) admission diagnosis; e) type of administered antimicrobial and other agents since hospital admission; and f) underlying comorbidities and predisposing illnesses. Using the available information, the Charlson’s Comorbidity Index (CCI) was calculated for each enrolled patient [19]. All this information was recorded by the investigators in one case report form.

### Laboratory assessments

A minimal volume of 5 ml of liquid stool was sampled from every enrolled patient with diarrhea, and transported in a sterile box within 30 minutes to the microbiological laboratory of the same hospital. Sampled stool was tested for the presence of *C*. *difficile* using the C. DIFF QUIK CHEK COMPLETE^®^ kit (Alere/TechLab, Blacksburg, USA) according to manufacturer’s instructions. Samples positive for glutamate dehydrogenase (GDH) and for either toxin A or toxin B were considered positive [20]. Samples yielded positive for the presence of GDH but negative for toxins were stored at -70°C, and then were transported to a central lab for the detection of *tcdA*, *tcdB* and *tcdC* genes by real-time PCR (Xpert^TM^, Adecco, New Zealand) or culture. Samples positive for *tcdA* and *tcdB* or yielding *C*. *difficile* at culture were also considered positive for a diagnosis of CDI [21].

### Study endpoints and objectives

The primary study endpoint was the point-prevalence of CDI in hospitalized patients in Greek hospitals. The point-prevalence of CDI was expressed as the number of patients with CDI per 10,000 patient bed-days (synonymous with occupied beds) as proposed elsewhere [2]. Two secondary variables of the point-prevalence of CDI were also estimated: a) the in-hospital diarrhea point-prevalence defined as the number of patients with diarrhea divided by the total number of screened patients; and b) the point-prevalence of CDI among patients with diarrhea defined as the number of patients with CDI divided by the total number of screened patients with diarrhea. Secondary study objectives were: a) to identify the risk factors associated with the development of CDI among cases with diarrhea; b) to identify the association of these factors with the time to development of CDI; and c) to propose a diagnostic score taking into consideration the CCI and clinical data parameters defined by logistic regression analysis. Another exploratory study endpoint was time to CDI. Since time to CDI is greatly influenced by risk factors of CDI, time to CDI was also analyzed with the secondary study endpoints.

### Sample size calculation

In order to calculate the sample size for correctly reporting the point-prevalence with its 95% confidence intervals (CI), the design effect (DEFF) was estimated [22]. Based on the number of hospitals to be included in the current study, the average hospital size, the total number of hospitals and the number of beds in acute care hospitals, DEFF was calculated to be equal to 4.5. Sample size calculations were made based on an expected point-prevalence of 5 cases per 10,000 patient bed-days. The reported point-prevalence could be estimated at a 95% confidence level given an overall sample size of 1536 subjects at each study period, provided that each hospital reported at least 50 cases.

### Statistical analysis

Per protocol, for the correct calculation of point-prevalence, it was absolutely necessary that the total number of occupied beds in each department at the specific date was reported. Departments not reporting this number were not included in the analysis. To identify risk factors for CDI in hospitalized cases with diarrhea, comparisons were done between patients with CDI and those without CDI at both study periods using chi-square or Fisher’s exact test for qualitative variables and Student’s t-test for quantitative variables; odds ratio (OR) and 95% CI were calculated by Mantel-Haenszel statistics. For predictors that are continuous, firstly ROC (Receiver Operating Characteristics) analysis was applied to investigate the discrimination ability on the secondary outcomes using values with cut-off greater than 85% for specificity. The validity of differences was confirmed by logistic regression analysis; OR and 95% CI were calculated. The time until development of diarrhea was calculated by subtracting the date of first presentation of in-hospital diarrhea as marked-up on patients’ file from the date of admission. The impact of the defined risk factors on the time until development of CDI-associated diarrhea was explored using Cox regression analysis; hazard ratios and 95% CI were determined. Kaplan-Meier analysis followed by log-rank test comparisons was also done for any variable proved significant after Cox regression analysis. In order to describe a prediction score for CDI, significant risk factors from logistic regression were added per patient to form a score. The score was analyzed by ROC curve analysis for its discrimination ability for CDI. Sensitivity, specificity, positive (PPV) and negative predictive value (NPV) of the best trade-off were calculated. Any two-tailed value of p below 0.05 was considered significant.

## Results

### Primary study endpoint

**Fig 1:**
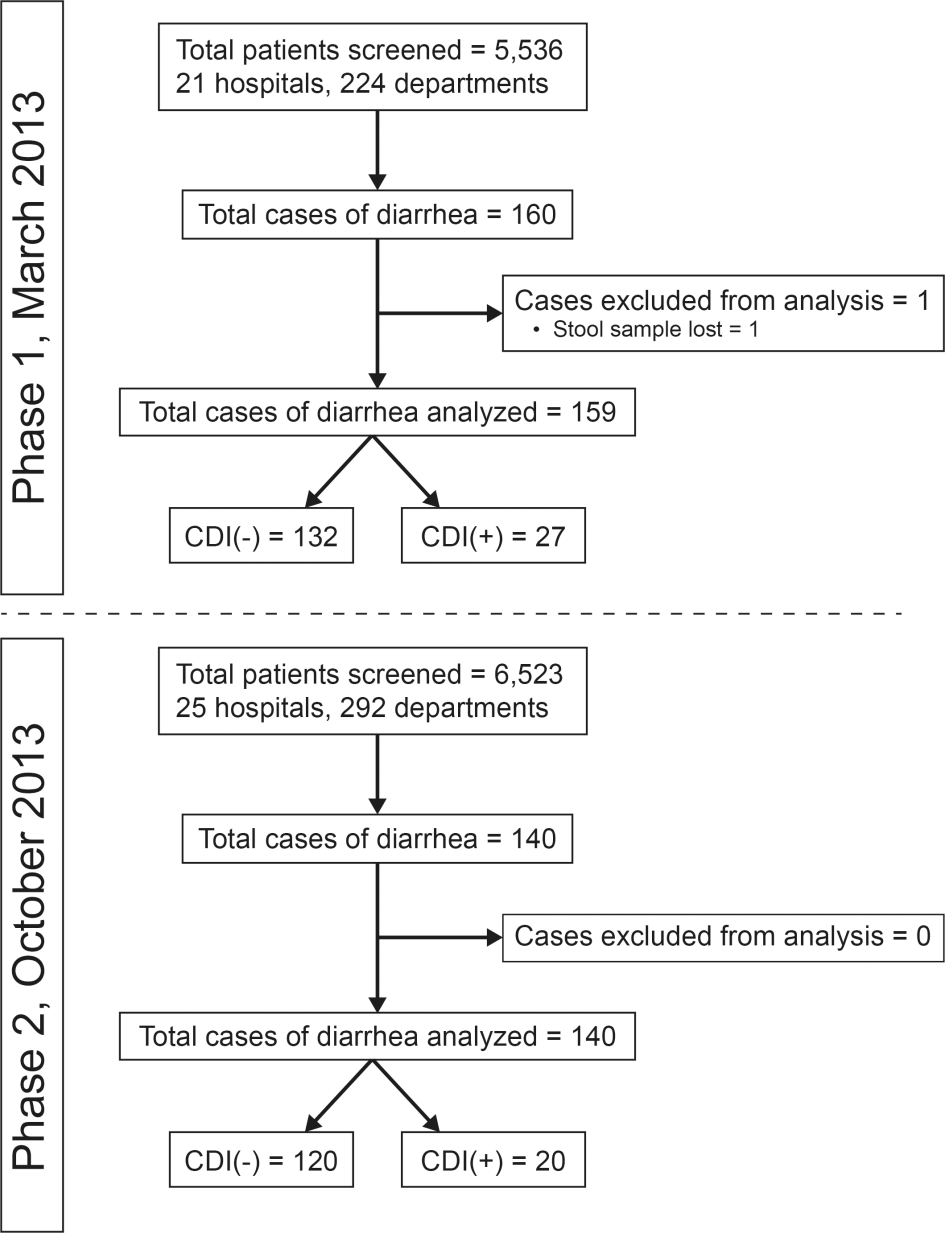
Study flow chart for each study period. CDI: *Clostridium difficile* infection

A total of 5,536 patients were screened during the first study period and 6,523 patients during the second study period. The study flow chart is shown in Fig 1. The point-prevalence of CDI in Greek hospitals was 5.6 per 10,000 patient bed-days in the first study period and 3.9 per 10,000 patient bed-days in the second study period (p = 0.111). The proportion of CDI among hospitalized patients with diarrhea was 16.98% in the first study period and 14.29% in the second period (p = 0.522 for the difference between the two study periods). Since these two estimates of the point-prevalence did not differ between the two study periods (Table 1), both periods were reported together. To this end, the overall point-prevalence of CDI in Greek hospitals was 4.6 per 10,000 patient bed-days (95% CI: 3.6–6.0) and the overall proportion of CDI among hospitalized patients with diarrhea was 15.7% (95% CI: 12.0–20.3%).

**Table 1:** Primary and secondary variables of point-prevalence of each phase of the study.

	Phase 1		Phase 2		p value of difference
		95% CI		95% CI	
Point-prevalence of CDI in Greek hospitals (per 10,000 patient bed-days)	5.6	3.8–8.3	3.9	2.5–6.2	0.111
**Point-prevalence of diarrhea in Greek hospitals (per 10,000 patient bed-days)**	**33.0**	**28.2**–**38.5**	**27.2**	**23.0**–**33.2**	**0.011**
Proportion of CDI among patients with diarrhea in Greek hospitals (per 100 patients with diarrhea)	16.98	11.94–23.58	14.29	9.16–21.45	0.522

Variables in bold indicate statistical significance.

### Secondary study objectives

Since the above indexes of point-prevalence did not differ between the two study periods, all 300 patients with diarrhea were analyzed together to explore risk factors related with the development of CDI. As one stool sample was lost, analysis comprised the remaining 299 cases of diarrhea. Among these cases, 47 were associated with a positive CDI diagnostic test. Comparisons between 47 patients with CDI and 252 patients without CDI are shown in Table 2. According to this analysis, the frequency of solid tumor malignancies, administration of antimicrobials after hospital admission, intravenous vancomycin or colistin administration after hospital admission and number of antimicrobials administered were significantly higher among CDI cases of diarrhea than in patients with diarrhea without CDI (p<0.05). The same analysis showed that CCI was significantly greater among patients with CDI than in patients without CDI. Using ROC analysis, it was found that a CCI >6 was accompanied by more than 80% specificity for CDI. More precisely, 14 patients with CDI (29.8%) had a CCI >6 compared to 33 (13.1%) patients without CDI (OR: 2.8, 95% CI: 1.4–5.8, p = 0.008). A similar coordinate point of the ROC curve to identify a precise cut-off number of antimicrobials administered as risk factor for CDI could not be found.

**Table 2:** Comparative characteristics of patients with diarrhea with and without *Clostridium difficile* infection (CDI).

	Patients without CDI (n = 252)	Patients with CDI (n = 47)	p
Male (number, %)	129 (51.2)	29 (61.7)	0.205
Age (mean ± SD, years)	66.9 ± 17.8	70.6 ± 16.5	0.187
Body temperature (mean ± SD, °C)	37.84 ± 0.79	37.73 ± 0.79	0.505
Unformed bowel movements (n, mean ± SD)	4.17 ± 2.19	3.57 ± 2.00	0.188
White blood cell count (mean ± SD, /mm^3^)	11630.8 ± 15427.7	11558.7 ± 7796.7	0.982
**Charlson’s Comorbidity Index (mean ± SD)**	**4.03 ± 2.32**	**5.35 ± 2.42**	**0.001**
Residence in long-term care facility (number, %)	8 (3.2)	3 (6.4)	0.388
On regular hemodialysis (n, %)	8 (3.2)	1 (2.1)	1.00
Nasogastric feeding tube (n, %)	26 (10.3)	7(14.9)	0.445
Inflammatory bowel disease (n, %)	11 (4.4)	1 (2.1)	0.699
Bone marrow transplantation (n, %)	1 (0.4)	2 (4.3)	0.065
Predisposing factors (n, %)			
Type 2 diabetes mellitus	52 (20.6)	14 (29.8)	0.181
Chronic heart failure	39 (15.5)	6 (12.8)	0.824
Chronic renal disease	25 (9.9)	7 (14.9)	0.308
Chronic obstructive pulmonary disorder	18 (7.1)	5 (10.6)	0.379
**Solid tumor malignancy**	**37 (14.7)**	**14 (29.8)**	**0.019**
Hematologic malignancy	22 (8.7)	7 (14.9)	0.186
Acute ischemic stroke	22 (8.7)	4 (8.5)	1.00
Acute hemorrhagic stroke	3 (1.2)	0 (0)	1.00
**Intake of antimicrobials (n, %)**	**194 (77.0)**	**43 (91.5)**	**0.030**
**Number of administered antimicrobials (mean ± SD)**	**1.96 ± 1.77**	**2.64 ± 2.12**	**0.017**
Beta-lactams/beta-lactamase inhibitors	32 (12.7)	7 (14.9)	0.642
Second-generation cephalosporins	23 (9.1)	6 (12.8)	0.425
Third-generation cephalosporins	25 (9.9)	7 (14.9)	0.308
Piperacillin/tazobactam	67 (26.6)	15 (31.9)	0.478
Fluoroquinolones	66 (26.2)	11 (23.4)	0.856
Aminoglycosides	26 (10.3)	9 (19.1)	0.089
Carbapenems	51 (20.2)	12 (25.5)	0.437
**Vancomycin**	**32 (12.7)**	**13 (27.7)**	**0.014**
Clindamycin	15 (6.0)	3 (6.4)	1.00
Macrolides	12 (4.8)	3 (6.4)	0.713
Metronidazole	65 (25.8)	16 (34.0)	0.283
Linezolid	14 (5.6)	1 (2.1)	0.480
Tigecycline	12 (4.8)	4 (8.5)	0.292
Daptomycin	16 (6.4)	3 (6.4)	1.00
**Colistin**	**26 (10.3)**	**10 (21.3)**	**0.048**
Intake of other drugs (n, %)			
Proton pump inhibitors	158 (62.7)	28 (59.6)	0.744
H_2_-blockers	23 (9.1)	6 (12.8)	0.425
Low-dose aspirin	30 (11.9)	5 (10.6)	1.00
Low-molecular weight heparin	97 (38.5)	17 (36.2)	0.870
Acenocoumarol	14 (5.6)	3 (6.4)	0.737
Corticosteroids	47 (18.7)	13 (27.7)	0.167
Chemotherapeutics	31 (12.3)	6 (12.8)	1.00
Non-steroidal anti-inflammatory drugs	16 (6.3)	4 (8.5)	0.532

Data of both study periods are reported together. Characteristics in bold indicate statistical significance.

The above four risk factors and a value for CCI >6 were entered into logistic regression analysis (Table 3). Results disclosed that solid tumor malignancy (OR: 2.69, 95% CI: 1.18–6.15, p = 0.019), intake of any antimicrobial after hospital admission (OR: 3.61, 95% CI: 1.03–12.76, p = 0.045), and intake of vancomycin (OR: 2.49, 95% CI: 1.09–5.64, p = 0.029) were independent risk factors related to CDI. CCI >6 exhibited a tendency to be an independent risk factor with an OR of 2.24 (95% CI: 0.98–5.10, p = 0.056). Similarly, colistin was not a significant independent factor.

**Table 3:** Logistic regression analysis of risk factors related to *Clostridium difficile* infection among patients with diarrhea.

	Odds ratio	95% confidence intervals	p
**Solid tumor malignancy**	**2.69**	**1.18–6.15**	**0.019**
**Intake of any antimicrobial**	**3.61**	**1.03–12.76**	**0.045**
**Intake of vancomycin**	**2.49**	**1.09–5.64**	**0.029**
Intake of colistin	1.84	0.76–4.43	0.173
Charlson’s Comorbidity Index >6	2.24	0.98–5.10	0.056

Factors in bold indicate statistical significance.

**Fig 2:**
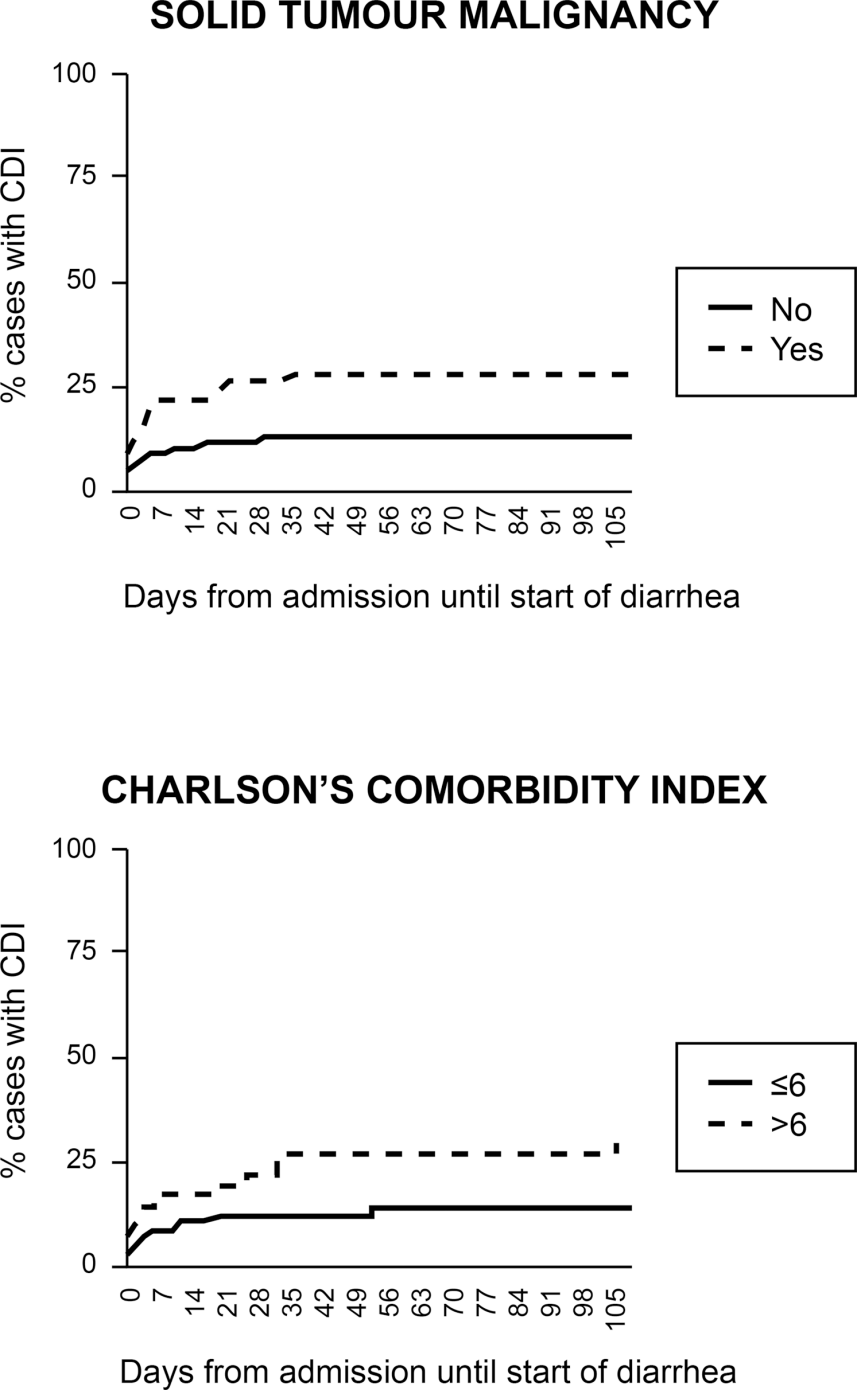
Impact of solid tumor malignancy and Charlson’s Comorbidity Index score more than 6 on the time until development of CDI.

Considering the time interval between hospital admission and onset of diarrhea as defined from each patient’s record, the impact of each of the above three independent risk factors and of CCI >6 on the time until presentation of CDI was explored. After censoring at 105 days as suggested previously [8], Cox hazard regression analysis was done to explore the influence of any of the four identified variables (Table 4). Analysis revealed solid tumor malignancy and CCI >6 to be the only variables associated with earlier presentation of CDI following hospital admission. The impact of these two variables on time to development of CDI is shown in Fig 2.

**Table 4:** Cox regression analysis of variables associated with time until development of CDI.

	Hazard ratio	95% confidence intervals	p
Solid tumor malignancy	2.37	1.17–4.78	0.016
Intake of any antimicrobial	2.06	0.71–5.93	0.181
Intake of vancomycin	1.79	0.89–3.56	0.099
Charlson’s Comorbidity Index >6	2.00	1.00–4.37	0.050

Next, a score was generated to explore the use of the above risk factors for predicting the diagnosis of CDI, i.e. solid tumor malignancy, intake of any antimicrobial, intake of vancomycin and CCI >6. Each of these risk factors scored equal in this score. Analysis revealed that the sensitivity, specificity, PPV and NPV for CDI with two or more of these risk factors was 61.7%, 70.6%, 28.2% and 90.8%, respectively. The OR for CDI with at least two of these factors was 3.88 (95% CI: 2.03–7.41, p<0.0001).

In our study, vancomycin was administered, likely intravenously, for infections other than CDI. However, this does not necessarily imply an association with an increased risk of developing CDI.

## Discussion

The C. DEFINE study is the first study to date dealing exclusively with CDI epidemiology in Greece in a large cohort of hospitalized patients (5,536 and 6,523 on the two sampling days). We revealed a considerable point-prevalence of CDI in Greek hospitals ranging between 3.9 and 5.6 per 10,000 patient bed-days, and between 14.3 and 17.0% of hospitalized patients with diarrhea, respectively.

The point-prevalence of CDI remained stable over the two time periods; however, the prevalence of diarrhea cases differed between the two study periods and this may be a reflection of the seasonal epidemiological variations.

The epidemiology of CDI is rapidly changing; the annual incidence is increasing globally [22] and this study presents not only the point-prevalence of CDI in hospitalized patients in Greece, but also provides evidence about the risk factors for CDI development that should be taken into consideration by attending physicians upon presentation of diarrhea. The majority of studies on CDI epidemiology have either been prospective, aiming to define the incidence of CDI over time, or retrospective analyses of case cohorts, aiming to disclose the risk factors related with CDI. A common denominator of these studies is that they explore the risk factors for CDI among the total enrolled patient population.

By contrast, the C. DEFINE study is unique in pinpointing the specific risk factors related with CDI when a patient presents with diarrhea. Indeed, this study could be used as a potential tool for clinicians, suggesting four main risk factors in hospitalized index patients with diarrhea: 1) presence of solid tumor malignancy; 2) administration of any antimicrobial agent after hospital admission; 3) intake of vancomycin after hospital admission; and 4) a CCI >6. Although no specific cut-off could be found for addition in the proposed risk factors of CDI, current findings definitively suggest that patients with CDI received significantly more antibiotics than patients without CDI. This finding is consistent with previous studies, which have identified the number of administered antibiotics as a risk factor for the development of CDI [8, 23].

Three recent publications report on the incidence of CDI both in the community and in hospital settings [24–26]. In the first study conducted from January to December 2012 in Barcelona, the incidence of CDI was 1.93 per 10,000 patient bed-days [24]. During the same time period, the incidence of CDI in Portugal was calculated 1.09 cases per 10,000 patient bed-days in most months of the year 2012. An outbreak was recorded in July 2012, leading to an increase of the incidence to 13.9 CDI cases per 10,000 patient bed-days [25]. Finally, in a 2-year study from August 2010 to July 2012 in Edinburgh, the incidence of community-acquired CDI was 6.4 per 100,000 patient-years and of healthcare-associated CDI 38.4 per 100,000 patient-years [26]. Although these estimates are comparable to those reported in the present study, it should be noted that they are indexes of incidence and not of prevalence. To our knowledge, only four studies have reported on the prevalence of CDI [2, 27–29]. The first reported on the yearly rate of CDI in hospitalized patients in USA. This was 5.21 per 1,000 patients in 2001, which steadily increased to 7.83 per 1,000 patients in 2010, similar to the prevalence reported in the present study [27]. The second study was a point-prevalence study of the asymptomatic carriage of toxigenic *C*. *difficile* in the stool of 160 hospitalized patients in Cleveland; this was found to be 18% [28]. The third study was conducted in Spain on a single day in patients aged ≥2 years. A total of 870 specimens from 730 patients were selected from 118 laboratories. The estimated rate of hospital-acquired CDI was 2.4 cases per 1,000 admissions or 3.8 cases per 10,000 patient-days [29]. The most recent study was a biannual point-prevalence study of CDI in hospitalized patients with diarrhea across Europe. A mean of 7.0 cases of CDI per 10,000 patient bed-days was found in the two study periods [2].

All studies assessing the incidence rates of CDI, retrospective studies and meta-analysis recognized recent intake of antibiotics as a major predisposing factor for the development of CDI in hospitalized population [7, 8, 23, 24]. Although, a recent meta-analysis suggests that almost all classes of antimicrobial agents are culprits [30], fluoroquinolones, third-generation cephalosporins, beta-lactam/beta-lactamase inhibitors combinations, and vancomycin have been reported as major triggers of CDI [8]. The risk for the development of CDI is higher when the time of exposure to antimicrobials is more than 18 days [8]. In the present study, intake of antimicrobials was also recognized as a risk factor for CDI. However, among antimicrobials, vancomycin was explicitly involved as attested by both logistic regression analysis and Kaplan-Meier analysis of the first 30 days after hospital admission. This indicates that recent exposure to vancomycin is an independent association for CDI [8].

Studies exploring the incidence of CDI and of recurrent CDI among hospitalized patients also report on the importance of administration of proton pump inhibitors [7, 25]. This was not confirmed in the present study because adjustments for comparisons were not done with the overall study population as in previous publications, but only with the other cases of diarrhea.

The main limitations of the present study were the lack of prospective design and the lack of uniformity of the study sites between the two study periods. However, the point-prevalence of CDI did not differ between the two periods of the study, providing robust results for the risk factors associated with CDI. It was also interesting to highlight the significance of solid tumors but not of hematologic malignancies as risk factor for CDI. This may be explained by the low number of studied cases with diarrhea and hematologic malignancies. In conclusion, our study shows a considerable point-prevalence of CDI in Greek hospitals. Major risk factors for CDI in diarrhea cases are intake of antimicrobials and in particular vancomycin administration, presence of solid tumor malignancy and CCI of >6. These results underline the importance of obtaining a timely and prompt diagnosis of CDI in hospitalized patients with diarrhea and could provide key information to support physicians in preventing CDI development in hospitalized patients.
